# Melanopsin Photopigment Comes in Two Distinct Forms

**DOI:** 10.1371/journal.pbio.0040263

**Published:** 2006-07-25

**Authors:** Mason Inman

Photoreceptors aren’t just for seeing. Deep within the retina of the vertebrate eye are photoreceptors that capture light to regulate non-visual processes such as day–night rhythms and the narrowing and widening of the pupil. To capture light, these special cells—called intrinsically photoreceptive retinal ganglion cells—require pigments called melanopsins, similar to the opsins that the rod and cone cells use for turning light into vision. While the rod and cone opsin proteins have been extensively studied, much less is known about the melanopsins, although they appear to function more like the opsins found in invertebrate eyes.

Now a new study shows that there are actually two distinct versions of melanopsin. The two genes and their associated proteins have persisted through hundreds of millions of years of evolution—only for one to be lost in the lineage leading to mammals. The discovery, by James Bellingham, Robert Lucas, and colleagues, clears up one set of questions, about the conservation of melanopsin genes through evolution. But it also opens up new questions about how the two related melanopsins function and why mammals came to lose one of them.

The first known melanopsin gene was discovered in the African clawed frog, *Xenopus laevis*. Comparing the sequence of the frog version of the gene with that of humans and mice, it appeared that the gene had been poorly conserved through evolution. The central core regions (the area most critical for function) of the *Xenopus* and human melanopsin proteins are only 55% identical, whereas the corresponding portions of their rod opsin sequences are 85% identical. This posed a conundrum: even though their sequences differed, the melanopsins were somehow related to each other, and seemed to have an important function in these diverse organisms.

Bellingham and colleagues found that the *Xenopus* and human melanopsins had been distinct for eons. The researchers trawled databases, and identified and sequenced new melanopsin genes from a wide variety of animals—including the African clawed frog, the zebrafish, and the chicken—to reconstruct the melanopsin family tree. They found that most vertebrates carry two versions of melanopsin, one similar to that originally found in *Xenopus* and the other more similar to the human gene. Bellingham and colleagues called these two versions of melanopsin Opn4x, after *Xenopus*-like, and Opn4m, for mammal-like. They bolstered the case for two lineages of melanopsins by looking at the chromosomes that carry the different versions of melanopsin genes, and at the genes that sit alongside the melanopsin genes. Importantly, this also revealed that mammals have lost the *Opn4x* gene during their evolution, leaving them unusual among the vertebrates in having only a single melanopsin.

With the revised lineage, it is clear that each version of melanopsin was fairly well conserved through evolution—except for the loss of *Opn4x* in the lineage leading to mammals. For both *Opn4x* and *Opn4m*, the sequences around the core region had been at least 66% conserved. This is significantly higher than the conservation estimates from previous studies, and higher than the similarity between the two groups.

Since the researchers found two distinct versions of melanopsin in three classes of vertebrates—fish, amphibians, and birds—this suggests the two versions split early in vertebrate evolution, before their ancestors came onto land, about 360 million years ago. Similarly, the analysis suggests that the lineage leading to mammals lost *Opn4x* early on, perhaps even before the separation of placental and marsupial mammals. Bellingham and colleagues also showed that the Opn4m of chickens functions as a sensory photopigment, as had been shown before for human and mouse melanopsins, confirming that the function of this gene has been conserved over evolutionary time.

So far, it’s not clear how the functions of the two versions of melanopsin differ. Maintaining separate cone opsin genes allows animals to sample different wavelengths of light and forms the basis of color vision. Could the two melanopsins underlie a similar quality for non-visual light detection? The researchers hope to answer this question by looking at the two melanopsin proteins of non-mammals in more depth. This will also reveal the implications for mammals of having only one melanopsin. Mammals also lost other photoreception proteins early in their evolution, leaving their color vision less sophisticated than that of birds, reptiles, fish, and amphibia. The loss of Opn4x reveals another way in which our experience of the light environment may be impoverished.

## 

**Figure:**
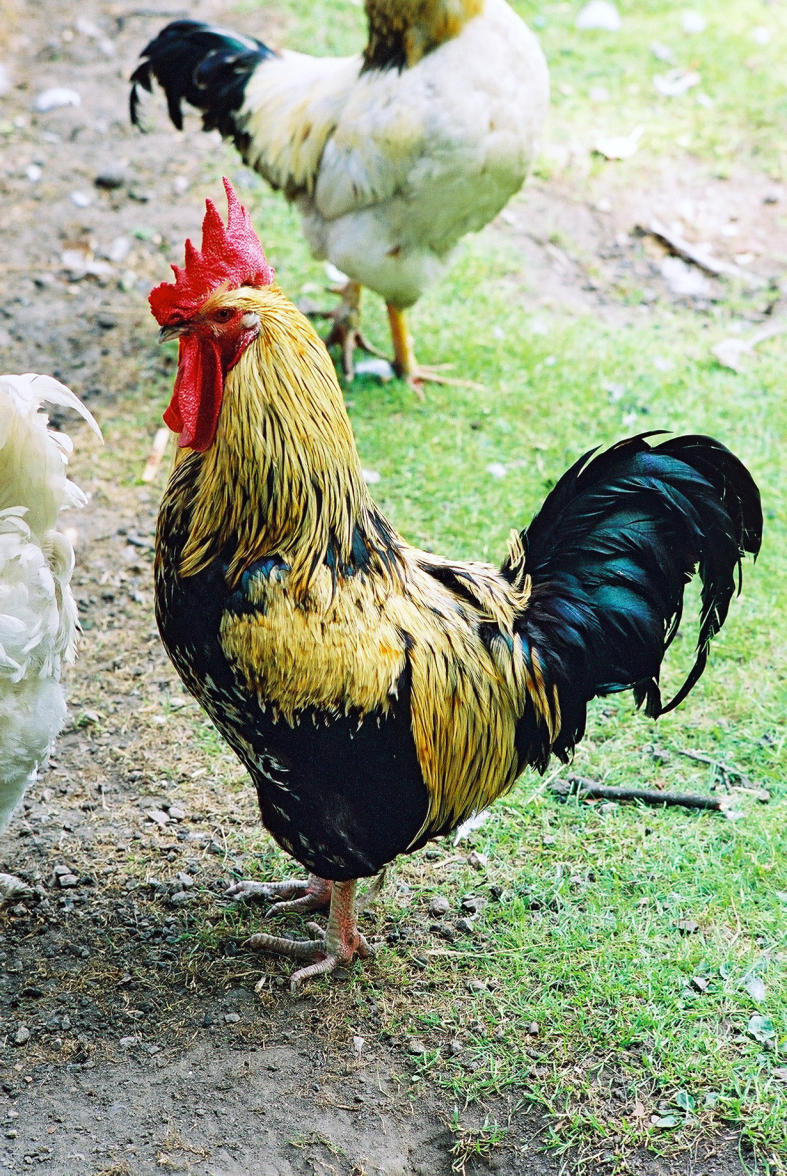
The eye of this chicken possesses two melanopsin photopigments whereas the human eye has only one melanopsin.

